# Non-syndromic premature ovarian insufficiency associated with monoallelic *LIG4* mutation via haploinsufficiency

**DOI:** 10.1186/s13048-026-02163-1

**Published:** 2026-06-24

**Authors:** Sha Yu, Xingyu Zhou, Yunhui Lai, Shuyan Tang, Shiling Chen

**Affiliations:** 1 Department of Gynecology, Shenzhen Second People’s Hospital, The First Affiliated Hospital of Shenzhen University Health Science Center, Shenzhen, 518000 China; 2 Center for Reproductive Medicine, Department of Gynecology and Obstetrics, Nanfang Hospital, Southern Medical University, No 1838 Guangzhou Northern Road, Guangzhou, 510515 China; 3 International Peace Maternity and Child Health Hospital, Shanghai Jiao Tong University School of Medicine, Shanghai, 200030 China; 4 Shanghai Key Laboratory of Embryo Original Diseases, Shanghai, 200030 China

**Keywords:** Premature ovarian insufficiency, DNA ligase IV (LIG4), Non-homologous end joining (NHEJ), Haploinsufficiency, DNA damage response (DDR)

## Abstract

**Background:**

Premature ovarian insufficiency (POI) is a heterogeneous reproductive disorder, with genetic factors, particularly defects in DNA damage response pathways, increasingly implicated in its pathogenesis. DNA ligase IV (LIG4) is a key enzyme in the non-homologous end joining (NHEJ) pathway responsible for repairing DNA double-strand breaks (DSBs). However, its role in non-syndromic POI remains unclear. This study aimed to investigate the potential contribution of *LIG4* variants to non-syndromic POI.

**Results:**

Whole-exome sequencing identified a heterozygous frameshift variant in *LIG4* (c.1271_1275del) in a three-generation Han Chinese family with non-syndromic POI, which co-segregated with affected individuals. AlphaFold-based structural modeling predicted truncation of the C-terminal XRCC4 interaction region. Functional experiments demonstrated that the mutant LIG4 protein showed reduced stability and was predominantly mislocalized to the cytoplasm of cells. In ovarian KGN cells, *LIG4* depletion reduced cell viability, induced stress-associated cellular senescence, and impaired DNA damage repair capacity. In *LIG4* knockout 293T cells, co-transfection of wild-type and mutant constructs revealed dose-dependent functional impairment, resulting in increased apoptosis under basal conditions and after phleomycin induced DNA damage, together with delayed repair of DSBs. Reanalysis of public single-cell RNA sequencing data further showed stage specific upregulation of *LIG4* during oocyte maturation. Co-expression network analysis revealed enrichment in the Fanconi anemia pathway, phosphatidylinositol 3-kinase signaling pathway, and glycan metabolism.

**Conclusions:**

Our findings suggest that monoallelic *LIG4* mutations may represent a potential genetic etiology for non-syndromic POI with sex-limited penetrance. While further validation in more physiologically relevant models is warranted, our data indicate that *LIG4* haploinsufficiency may impair DSB repair and disrupt molecular pathways crucial for oocyte maturation and survival, highlighting a potential role of the NHEJ pathway in maintaining human ovarian function.

**Supplementary Information:**

The online version contains supplementary material available at https://doi.org/10.1186/s13048-026-02163-1.

## Introduction

Premature ovarian insufficiency (POI), defined as the cessation of ovarian function before the age of 40 years, affects 3.7% of women globally during their reproductive years [1] and poses risks to fertility and health [1]. While the etiology encompasses autoimmunity, infections [2], and iatrogenic factors, the majority of cases remain idiopathic [3], with a strong heritable component. The landscape of POI genetics has expanded significantly with the widespread application of whole-exome sequencing (WES). To date, over 100 causative genes have been identified, clustered in critical pathways such as meiosis and DNA repair (e.g., *STAG3*, *MSH4*, *and HFM1*), follicle development (e.g., *GDF9* and *BMP15*), and mitochondrial function [4–7]. Large-scale cohort studies have shown that these known genes account for only 20–25% of cases [8], indicating that numerous pathogenic variants remain unidentified.

Emerging genetic evidence highlights the vulnerability of the ovaries to DNA damage. Genome-wide- association studies (GWAS) have linked variants in more than 50 DNA damage response (DDR) genes to the age at natural menopause [9], suggesting that DDR pathways are key determinants of ovarian lifespan. Mechanistically, DDR networks govern the entire trajectory of the ovarian reserve, from its establishment to eventual depletion. Efficient repair mechanisms are required at every stage: safeguarding the rapid proliferation of primordial germ cells (PGCs) in the embryo, resolving programmed double-strand breaks (DSBs) during meiotic recombination, and maintaining genomic stability in oocytes that remain dormant for decades [10, 11]. Accumulation of DNA damage and defective DDR not only compromise oocyte genomic integrity, leading to meiotic failure and apoptosis, but also induce senescence in granulosa cells (GCs), which is a key pathogenic process in POI [12].

Among the DDR pathways, non-homologous End Joining (NHEJ) is particularly relevant to POI because it repairs DSBs in both somatic and germ cells. Central to the NHEJ pathway is DNA ligase IV (LIG4), which forms a complex with XRCC4 to execute the final ligation step in DSB repair [13]. The LIG4-XRCC4 complex is essential for genomic stability and V(D)J recombination during lymphocyte development [14]. Historically, biallelic *LIG4* mutations have been associated with LIG4 syndrome, a rare autosomal recessive disorder characterized by severe combined immunodeficiency, microcephaly, and growth retardation [15, 16]. Owing to the severity of these biallelic phenotypes, the potential reproductive impact of heterozygous *LIG4* loss-of-function (LoF) variants has long been overlooked. However, recent reports indicate that monoallelic LIG4 variants can drive isolated immune dysregulation via haploinsufficiency [17], challenging the assumption that heterozygosity is benign. Together with the observation that haploinsufficiency of another core NHEJ factor, *NHEJ1*, can cause non-syndromic POI in humans and mice [18], these findings raise the possibility that subtle reductions in *LIG4* dosage may similarly compromise ovarian function without causing overt systemic syndromic features.

In this study, we aimed to determine whether monoallelic *LIG4* variant contribute to non-syndromic POI. Using WES in a Han Chinese family, we identified a novel heterozygous LoF variant of *LIG4* that co-segregated with the POI phenotype. To explore the underlying mechanism, we combined structural modeling with in vitro functional assays, demonstrating that the mutant protein was unstable and impaired overall DNA repair capacity, thereby accelerating cellular senescence under genomic stress. Furthermore, reanalysis of single-cell transcriptomic data mapped the spatiotemporal expression of *LIG4* during folliculogenesis, highlighting its critical role in oocyte maturation. Collectively, our findings provide evidence that *LIG4* haploinsufficiency represents a novel genetic etiology for non-syndromic POI, driven by compromised DNA repair efficacy and heightened ovarian vulnerability to genomic damage.

## Methods

### Study individuals and ethical approval

Female patients with POI were recruited from Shenzhen Second People’s Hospital (Shenzhen, China). The inclusion criteria were amenorrhea/oligomenorrhea for at least 4 months before the age of 40 years, with abnormal serum follicle-stimulating hormone (FSH) levels (> 25 IU/L) [19]. All affected individuals were evaluated and excluded for chromosomal abnormalities, ovarian surgery, chemotherapy, radiotherapy, and autoimmune diseases, including systemic lupus erythematosus, Sjögren’s syndrome, rheumatoid arthritis, and autoimmune thyroiditis. All patients had normal karyotypes (46, XX).

### DNA extraction and WES

Total genomic DNA was extracted from all participants using a MagPure Buffy Coat DNA Midi KF Kit (MAGEN, Shanghai, China) according to the manufacturer’s protocol. Samples from the proband and her elder sister were subjected to WES using an MGISEQ-2000 sequencing platform (BGI, Shenzhen, China). WES processing and data analysis were performed as previously described [20]. Raw data of 10 Gb per exome were mapped to a human reference genome (GRCh37/hg19) using Burrows-Wheeler Alignment. Variant calling was performed using the Genome Analysis Toolkit. Variants with minor allele frequencies ≥ 0.001 according to the 1000 Genomes Project or ExAC databases were excluded, and only those in coding regions or splicing sites were preserved. Missense variants predicted to be deleterious by in silico tools and LoF variants were selected as candidates. Variants in genes that are functionally related to ovarian development were preferred.

### Mutation validation and co-segregation analysis

Sanger sequencing was performed to validate the candidate variant identified by WES and to evaluate its co-segregation with the POI phenotype in the family. Specific PCR primers were designed using Primer Premier 5 (http://www.pre-mierbiosoft.com/primerdesign/). The sequences of *LIG4* primers used were *LIG4*-F: 5’-GATGGTGAGATGATGGCCTA − 3’and *LIG4*-R: 5’-CTGCTTGGTGGAGCTTTTCT-3’. Primers for *LIG4* were synthesized, and the PCR products were sequenced by BGI (Shenzhen, China).

### Bioinformatics analysis: structural prediction of the LIG4 wild-type and mutant protein

The physicochemical properties of the wild-type and mutant proteins were predicted using the ProtParam tool (https://web.expasy.org/protparam). Domain annotation of LIG4 protein was based on UniProt (P49917) and InterPro 104.0 (https://www.ebi.ac.uk/interpro) [21], and the domain architecture was visualized using the IBS web server [22]. Secondary structure predictions were performed using SOPMA [23] (https://npsa-prabi.ibcp.fr) and PSIPRED (http://bioinf.cs.ucl.ac.uk/psipred/). The three-dimensional structures of wild-type and mutant LIG4 and LIG4-XRCC4 complexes were modeled using AlphaFold 3 [24].

### Plasmids construction

Overexpression plasmids encoding wild-type (WT) and mutant (MT) human *LIG4* were constructed by GenomediTech (Shanghai, China). The lentiviral expression vector PGMLV was used as the backbone. The full-length human *LIG4* coding sequence (CDS) and mutant CDS with c.1271_1275del deletion were amplified by PCR using primers with 5’ homologous overhangs. Because the *LIG4* mutation causes C-terminal truncation, a 3×Flag tag was fused to the N-terminus. The PGMLV vector contains AmpR, ZsGreen, and puromycin resistance genes. All plasmids were verified using Sanger sequencing.

### Generation of the *LIG4-*knockout cell line

*LIG4*-knockout 293T cells were generated using the CRISPR/Cas9 system (Genomeditech, Shanghai, China). Briefly, a chemically synthesized sgRNA targeting the human *LIG4* gene (5′-ATTAAACCAGAGTATGTCAG-3′) was complexed with Cas9 nuclease and delivered to 293T cells by electroporation. Editing efficiency was confirmed by Sanger sequencing of pooled cells 72 h post-electroporation. Single clones were isolated by limiting dilution and genotyped by Sanger sequencing, followed by TA cloning. A *LIG4*^−/−^ clone carrying a 1-bp insertion on one allele and a 10-bp deletion on the other, both resulting in frameshift mutations, was selected for subsequent experiments.

### Cell culture and RNA interference or plasmid transfection

HEK293T and *LIG4*-KO 293T cells were cultured in DMEM (Keycell Biotechnology). KGN cells were cultured in DMEM/F12 (Keycell Biotechnology). Small interfering RNAs (siRNAs) were purchased from Genomeditech (Shanghai, China). For functional assays, cells were transfected with wild-type or mutant *LIG4* plasmids or siRNAs using Lipofectamine™ 2000 (Invitrogen) according to the manufacturer’s protocol. Cells were then treated with phleomycin (Sangon Biotech) for the indicated duration and harvested at the indicated time points.

### RT-qPCR, western blotting and immunofluorescence analyses

Total RNA extraction and reverse transcription were performed using TRIzol and HiScript II Select qRT SuperMix (Vazyme), followed by qPCR using AceQ qPCR SYBR Green Master Mix (Vazyme), according to the manufacturer’s instructions. RT-qPCR, western blotting, and immunofluorescence analyses were performed as described previously [25]. The sequences of primers used are as follows: human *GAPDH* forward 5′-TCAAGAAGGTGGTGAAGCAGG-3′, *GAPDH* reverse 5′-TCAAAGGTGGAGGAGTGGGT-3′; and human *LIG4* forward 5′-GAGATGACAAGGAGTGGCA-3′, *LIG4* reverse 5′-AGGCTTTGGCTGGCTATC-3′. The primary antibodies used were as follows. Western blotting: FLAG (66008-4-Ig, Proteintech; 1:5000), LIG4 (12695-1-AP, Proteintech; 1:500), 53BP1 (NB100-304SS, Novus; 1:500), γH2AX (9718T, CST; 1:1000), and GAPDH (60004-1-Ig, Proteintech; 1:5000). Immunofluorescence: γH2AX (9718T, CST; 1:100), 53BP1 (NB100-304SS, Novus; 1:100), and FLAG (66008-4-Ig, Proteintech; 1:100).

### CCK-8 assay, SA-β-gal staining and flow cytometry

KGN cell viability and senescence were assessed using the Enhanced CCK-8 assay (Keycell Biotechnology) and SA-β-gal staining (Beyotime), respectively. Apoptosis in *LIG4*-KO 293T cells was analyzed using an Annexin V-APC/7-AAD detection kit (Keycell Biotechnology). All assays were performed according to the manufacturer’s instructions. The treatment conditions are described in results and the figure legends.

### Single-cell RNA sequencing dataset reanalysis

Single-cell transcriptome data of human folliculogenesis were obtained from the GEO database (GSE107746) [26], comprising 83 oocytes and 92 granulosa cells across five follicular stages (primordial, primary, secondary, antral, and preovulatory follicles), with gene expression reported as log₂(FPKM + 1). Downstream analyses were conducted based on the quality control and cell annotations provided by the original study. *LIG4* expression was first compared between oocytes and granulosa cells at each developmental stage. Co-expression analysis was then performed in oocytes by calculating the Pearson correlation coefficients between *LIG4* and all coding genes within each stage. Genes showing significant positive correlations (*P* < 0.05, *r* > 0) in at least three stages and a global trend correlation > 0.8 across the five-stage expression trajectory were retained as *LIG4* co-expressed genes. Gene Ontology (GO) and The Kyoto Encyclopedia of Genes and Genomes (KEGG) enrichment analyses were performed on the resulting gene set, with adjusted *P* < 0.05 as the significance threshold.

### Statistical analysis

Statistical analyses and data visualization were primarily performed using GraphPad Prism software (version 10.6.1). Bioinformatics analyses and associated visualizations were conducted using R software (version 4.2.1) and Python (version 3.10.16). Continuous variables were first assessed for normality using the Shapiro-Wilk test. Data are presented as mean ± SEM. For comparisons between two groups, the unpaired Student’s *t*-test was used for normally distributed data, while Welch’s *t*-test was applied when the variances were unequal. For comparisons among three or more groups, the homogeneity of variance was first verified using the Brown-Forsythe test. For data with equal variances, ordinary one-way analysis of variance (ANOVA) followed by Tukey’s multiple comparison test was performed. In cases of unequal variances, Welch’s ANOVA followed by Dunnett’s T3 multiple comparison test was used. Differences in *LIG4* transcriptomic expression were analyzed using two-way ANOVA, followed by Sidak’s multiple comparisons test. All statistical tests were two-tailed, and a *P*-value of < 0.05 was considered statistically significant.

## Results

### Co-segregation of a heterozygous LoF variant of *LIG4* with POI in a Chinese family

**Fig. 1:**
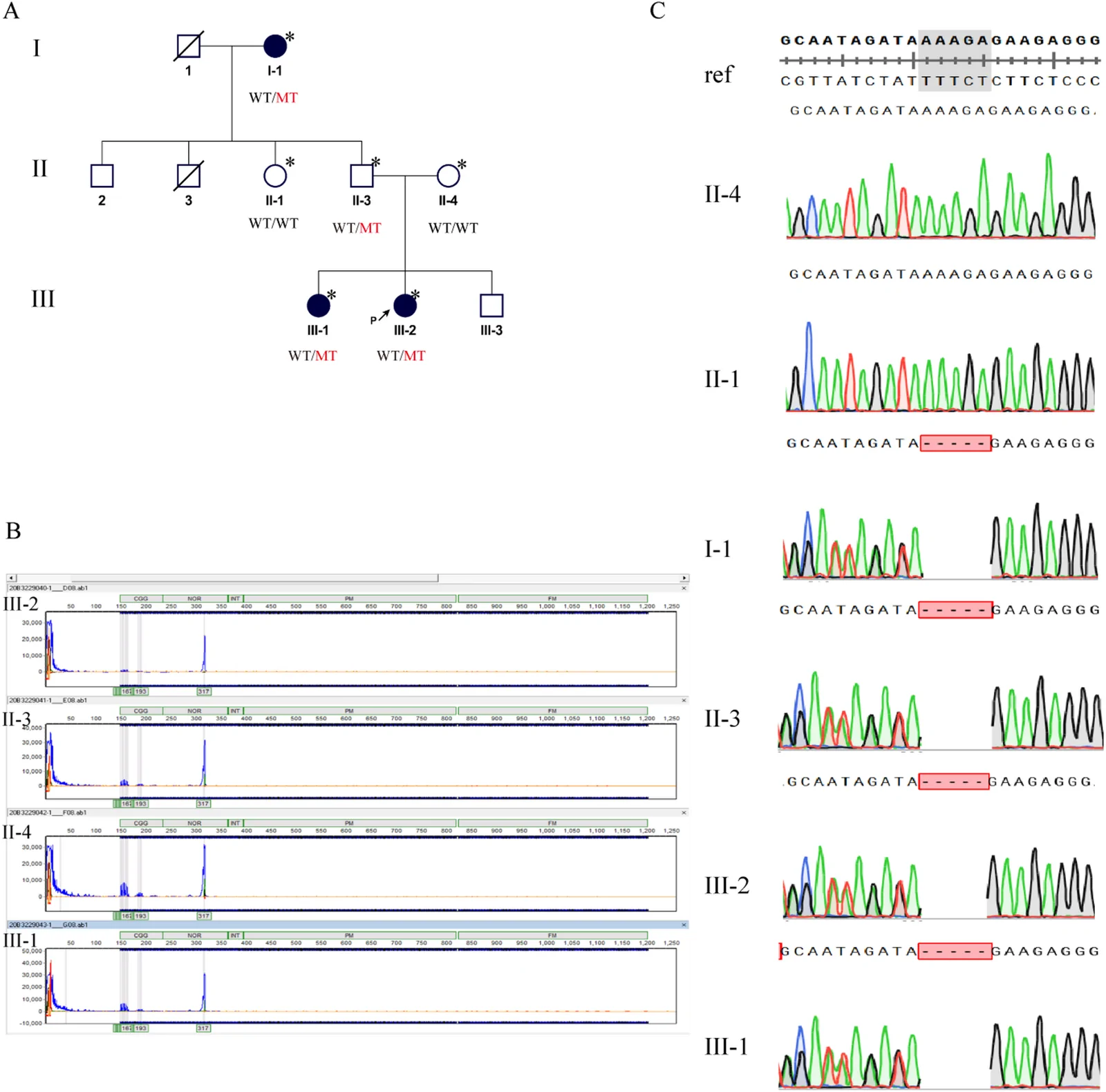
Pedigree and genetic analysis of a Chinese Han family with POI. **A** Pedigree of the family. The proband is indicated by an arrowhead. Affected females are shown as filled circles, and individuals subjected to Sanger sequencing are marked with asterisks. **B***FMR1* CGG repeat analysis. **C** Sanger sequencing results of the candidate variant in family members, as indicated in the pedigree

We enrolled a Han Chinese family with POI (Fig. 1A). The proband (Ⅲ-2) was diagnosed with POI at 31, following 2 years of primary infertility after marriage (Table 1). Her sister (Ⅲ-1) was fertile, married at 24, delivered two girls at 24 and 27, had one induced and one missed abortion, and delivered a boy by cesarean at 31. She was diagnosed with POI at 33. The proband’s parents (Ⅱ-3 and Ⅱ-4) were healthy, lived in Zhoukou, Henan Province, China, and were not consanguineous. The mother (Ⅱ-4) experienced menopause at 53. She had two half-brothers with normal fertility. The proband’s aunt had two children, experienced menopause after 50, and remained healthy.

**Table 1:** Clinical and hormonal characteristics of the two affected sisters

Characteristic	Proband III-2	III-1
Age at POI diagnosis (years)	31	33
First menses (years)	13	13
Height (cm)	155	163
Weight (kg)	54	60
FSH (mIU/ml)	30.63	80.56
LH (mIU/ml)	14.15	34.75
Estradiol (pg/ml)	61.90	23.91
Progesterone (ng/ml)	< 0.05	0.50
AMH (ng/ml)	0.22	0.08
Karyotype	46, XX	46, XX
*FMR1* CGG repeats	29/29	29/29

The proband’s grandmother (I-1) was over 80 and had amenorrhea at 39, which was considered consistent with a diagnosis of POI. She had four children (three sons and one daughter), all of whom had normal fertility. The pedigree is shown in Fig. 1A. No family members had intellectual disabilities, microcephaly, developmental delay, immunodeficiency, anemia, lymphopenia, or other systemic abnormalities.

To exclude X-linked causes of POI, we first performed *FMR1* CGG-repeat analysis in individuals II‑3, II‑4, III‑2, and III‑1, and no premutation or full mutation alleles were detected (Fig. 1B). We then performed WES on the two affected sisters (III‑2 and III‑1). Only variants shared by the two individuals were prioritized, leading to the identification of a heterozygous *LIG4* variant (c.1271_1275del, p. Lys424ArgfsTer20; NM_001098268.2) in both subjects III-2 and III-1. This variant was confirmed by Sanger sequencing (Fig. 1C). Segregation analysis showed that the proband’s father (II-3) and grandmother (I-1) also carried the same heterozygous LoF *LIG4* variant.

### Bioinformatics-based prediction of structure and pathogenicity of *LIG4* mutation

The *LIG4* frameshift variant c.1271_1275del is extremely rare in global population databases (ExAC allele frequency 0.000148; 1000 Genomes allele frequency 0; gnomAD allele frequency 0.000163). To comprehensively evaluate the background frequency of this variant in ethnically matched populations, we interrogated multiple major Chinese genomic databases. The variant was consistently ultra-rare across all independent cohorts: completely absent in the PGG.Han database, with allele frequencies of 0.000246 in the GDBIG cohort (*n* = 4,053), 0.000335 in the Westlake BioBank for Chinese (WBBC, *n* = 4,489), 0.000472 in the ChinaMAP project (*n* = 10,588), and 0.0005 in the NyuWa Genome Resource (*n* = 2,999). Furthermore, this variant is predicted to be highly deleterious (CADD score = 37) and is currently classified as Pathogenic in ClinVar. Together, these clinico-genomic features robustly meet the ACMG (American College of Medical Genetics and Genomics) criteria for pathogenicity, satisfying both PVS1 and PM2 evidence.

**Fig. 2:**
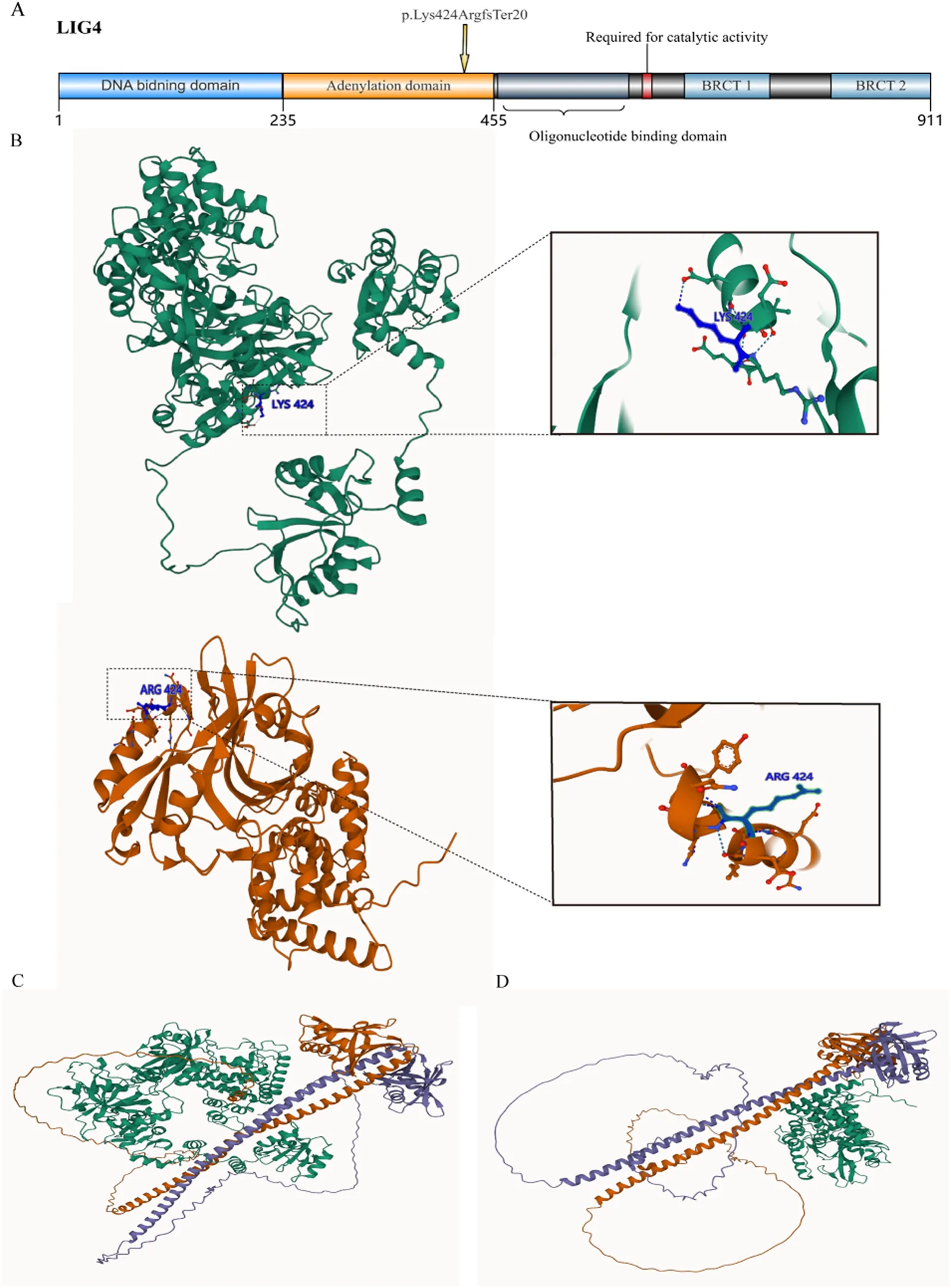
Structural analysis of wild-type and mutant LIG4 protein. **A** Schematic representation of the wild-type LIG4 protein primary structure. The yellow arrow indicates the frameshift mutation site in the adenylation domain. BRCT: BRCA1 C-terminal domain. **B** Structural modeling of the catalytic core of the complex. Wild-type LIG4 (green) shows Lys424 as blue sticks with a hydrogen-bond network (inset, blue dashed lines). The p. Lys424ArgfsTer20 mutant (orange) shows the substituted Arg424 residue (blue sticks) and its altered hydrogen-bond network involving the guanidinium group (inset). **C**–**D** Predicted tertiary structures of the LIG4-XRCC4 complex for wild-type (**C**) and p. Lys424ArgfsTer20 mutant (**D**) LIG4 (LIG4 in green; XRCC4 subunits in orange and purple)

The physicochemical properties of the wild-type and mutant LIG4 proteins are summarized in Table 2. The *LIG4* variant significantly alters the LIG4 protein’s physicochemical properties. LIG4 contains three domains (Fig. 2A): DNA-binding (DBD), oligonucleotide-binding (OBD), and adenylation/activation (AdD). AdD and OBD form the catalytic core. The C-terminus contains two BRCT (BRCA1 C-terminal repeat) domains, at residues 654–743 (BRCT1) and 808–911 (BRCT2). The C-terminal BRCT motifs and XRCC4-binding region are required for XRCC4 interactions. Bioinformatic analysis showed that the *LIG4* variant was a LoF mutation. At the DNA level, this variant has a five-nucleotide deletion spanning positions 1271–1275. The deletion causes a frameshift that substitutes Lys at position 424 with Arg, reconstitutes the downstream amino acid sequence, and introduces a premature termination codon 20 residues after the frameshift. This truncated protein lacks approximately the C-terminal half of LIG4, including both BRCT motifs and the XRCC4-interaction region.

**Table 2:** Physicochemical Properties of Wild LIG4 Protein and Mutant LIG4 Protein

Physicochemical properties	Wild LIG4	Mutant LIG4
Sequence length (number of amino acids)	911	443
Theoretical molecular weight (Da)	≈ 103,970.77	≈ 50,718.27
Theoretical isoelectric point (pI)	8.17	8.86
Number of positively/negatively charged residues (Arg + Lys / Asp + Glu)	126 / 121	61 / 54
Total number of atoms	14,635	7,146
Instability index (II)	38.01	36.83
GRAVY (hydrophobicity)	-0.399	-0.434
Aliphatic index	83.82	85.03

Secondary structure predictions (Figure S1) from both algorithms consistently indicated significant conformational remodeling of the mutant protein. Specifically, truncation abolishes the C-terminal BRCT domains and induces a shift in the remaining N-terminal architecture toward a more compact form, characterized by increased α-helix content (rising from 41.93% to 45.37% in SOPMA analysis) and decreased β-sheet elements. Notably, this remodeling involves the conversion of β-sheet regions within and downstream of the AdD into α-helices or random coils. These data suggest that the frameshift not only deletes functional C-terminal domains but also disrupts the local folding topology of the periphery of the catalytic core.

Structural modeling of wild-type LIG4 (Fig. 2B) revealed a complete multidomain architecture, where Lys424 is positioned within the catalytic core and maintains local structural integrity through a stabilizing hydrogen-bond network. In stark contrast, the p. Lys424ArgfsTer20 mutant (Fig. 2B) is truncated at the C-terminus, resulting in the complete loss of the OBD and tandem BRCT domains. This truncation induces a marked rearrangement of the remaining N-terminal fold, characterized by increased α-helical content and reduced β-sheet elements, consistent with secondary-structure predictions. At the atomic level, the substitution of Lys424 with arginine introduces a longer and more rigid side chain that alters local hydrogen bonding, indicative of a remodeled microenvironment.

Regarding the LIG4-XRCC4 interaction, the wild-type protein forms a well-defined complex with the XRCC4 homodimer (Fig. 2C), recapitulating known crystal structures: the LIG4 catalytic core engages the XRCC4 “head,” while the C-terminal BRCT domains anchor to the coiled-coil “stalk.” However, the mutant failed to establish stable contacts with XRCC4 because of the absence of BRCT modules (Fig. 2D). In the predicted model, the mutant protein remained largely dissociated from XRCC4, with the residual C-terminus appearing as a low-confidence disordered segment. These data confirm that the *LIG4* frameshift variant is a LoF variant that abrogates LIG4-XRCC4 assembly, thereby impairing NHEJ ligation.

### *LIG4* p. Lys424ArgfsTer20 is a loss-of-function allele, and *LIG4* knockdown impairs DSB repair and cellular fitness in KGN cells

**Fig. 3:**
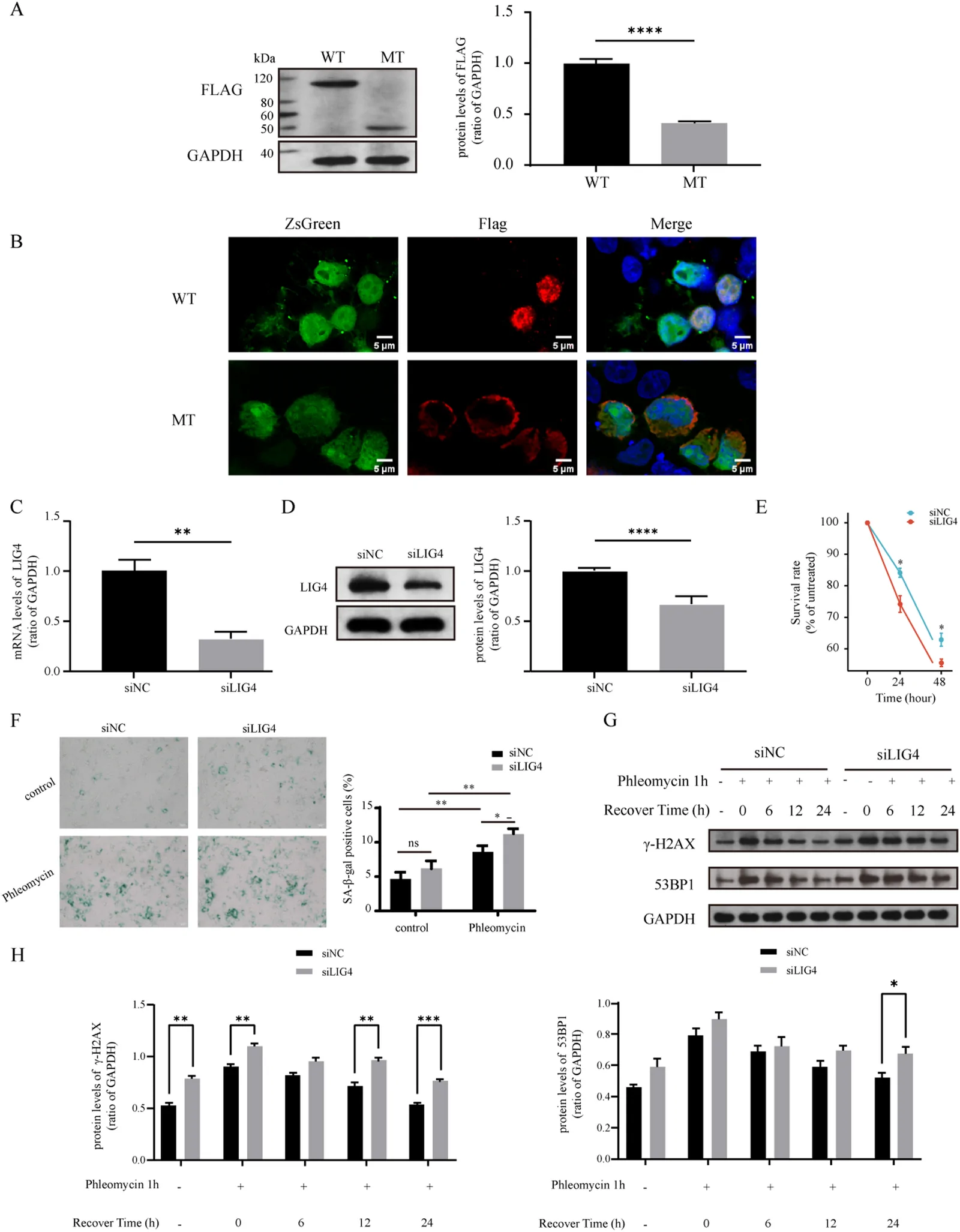
Monoallelic *LIG4* mutation impacts protein stability and localization, and *LIG4* knockdown impairs KGN cell viability and DNA damage repair. **A** Anti-FLAG immunoblots showing reduced abundance of the truncated mutant LIG4 (p. Lys424ArgfsTer20) compared to full-length wild-type (WT) FLAG-LIG4 in HEK293T cells (****P < 0.0001). **B** Immunofluorescence of HEK293T cells showing nuclear localization of WT LIG4 and cytoplasmic retention of MT LIG4 protein (FLAG, red; ZsGreen, green; DAPI, blue). Scale bar, 5 μm. **C**-**D** In KGN cells, *LIG4* siRNA (siLIG4) efficiently reduced *LIG4* mRNA (C, RT-qPCR) and protein levels (D, western blot) compared with negative control siRNA (siNC) (**P < 0.01, ****P < 0.0001). **E** After transfection for 48 h, KGN cells were treated with phleomycin (20 µg/mL); cell viability at 24 and 48 h was decreased in siLIG4 versus siNC (*P < 0.05). **F** Representative SA-β-gal staining and quantification in KGN cells transfected with siNC or siLIG4 for 48 h, with or without phleomycin (50 µg/mL, 1 h) and a 48 h recovery in fresh medium, showing enhanced DNA damage-induced senescence upon *LIG4* knockdown (scale bar, 50 μm; *P < 0.05, **P < 0.01; ns, not significant). **G**-**H** Western blot analysis of γH2AX and 53BP1 in KGN cells transfected with siNC or siLIG4. Cells were harvested either without phleomycin or after phleomycin treatment (50 µg/mL, 1 h), followed by medium replacement and recovery for 0, 6, 12, or 24 h. (****P < 0.0001, **P < 0.001, *P < 0.01, *P* < 0.05)

Overexpression of 3×Flag tagged *LIG4* (WT or MT) in HEK293T cells was confirmed by western blotting using an anti-FLAG antibody. A band corresponding to the full-length 3×Flag LIG4 protein (~ 107 kDa) was detected in cells expressing WT LIG4, whereas the p. Lys424ArgfsTer20 variant produced a markedly smaller band at ~ 53 kDa, consistent with a truncated product, and showed substantially reduced protein abundance compared with WT LIG4 (Fig. 3A). In line with these findings, immunofluorescence demonstrated predominant nuclear localization of WT LIG4, whereas the mutant protein was largely retained in the cytoplasm and was absent from the nuclei (Fig. 3B).

To evaluate the functional consequences of reduced *LIG4* dosage in a physiologically relevant context, we used KGN cells as an in vitro granulosa cell model and partially downregulated endogenous *LIG4* expression using siRNA. RT-qPCR and western blotting confirmed efficient knockdown of *LIG4* at both the mRNA (Fig. 3C) and protein levels (Fig. 3D). Following phleomycin challenge, CCK-8 assays revealed that siLIG4 cells exhibited significantly decreased viability compared to siNC cells at both 24 and 48 h (Fig. 3E). SA-β-gal staining showed no appreciable difference between the siLIG4 and siNC groups under basal conditions; however, after acute phleomycin-induced DNA damage, the proportion of SA-β-gal-positive cells increased in both groups, with a significantly greater increase in the siLIG4 cells (Fig. 3F). These findings indicate that reduced *LIG4* dosage compromises cellular fitness under genotoxic stress and exacerbates DNA damage-induced senescence in granulosa cells.

Given that *LIG4* knockdown aggravated DNA damage-induced senescence, we examined DSB repair kinetics. KGN cells transfected with siNC or siLIG4 for 48 h were either left untreated (baseline) or challenged with phleomycin (50 µg/mL for 1 h) and then allowed to recover in fresh medium for 0, 6, 12, or 24 h (Fig. 3G). Compared with siNC cells, siLIG4 cells displayed significantly higher γH2AX levels at 0, 12, and 24 h recovery time points. For 53BP1, no significant differences were detected at 0, 6, or 12 h; however, 53BP1 levels remained significantly elevated in the siLIG4 cells at 24 h (Fig. 3H). These data demonstrate that LIG4 knockdown impairs the DSB repair capacity of KGN cells.

### *LIG4* p. Lys424ArgfsTer20 is a haploinsufficient mutation with dose-dependent impairment of DNA repair capacity

**Fig. 4:**
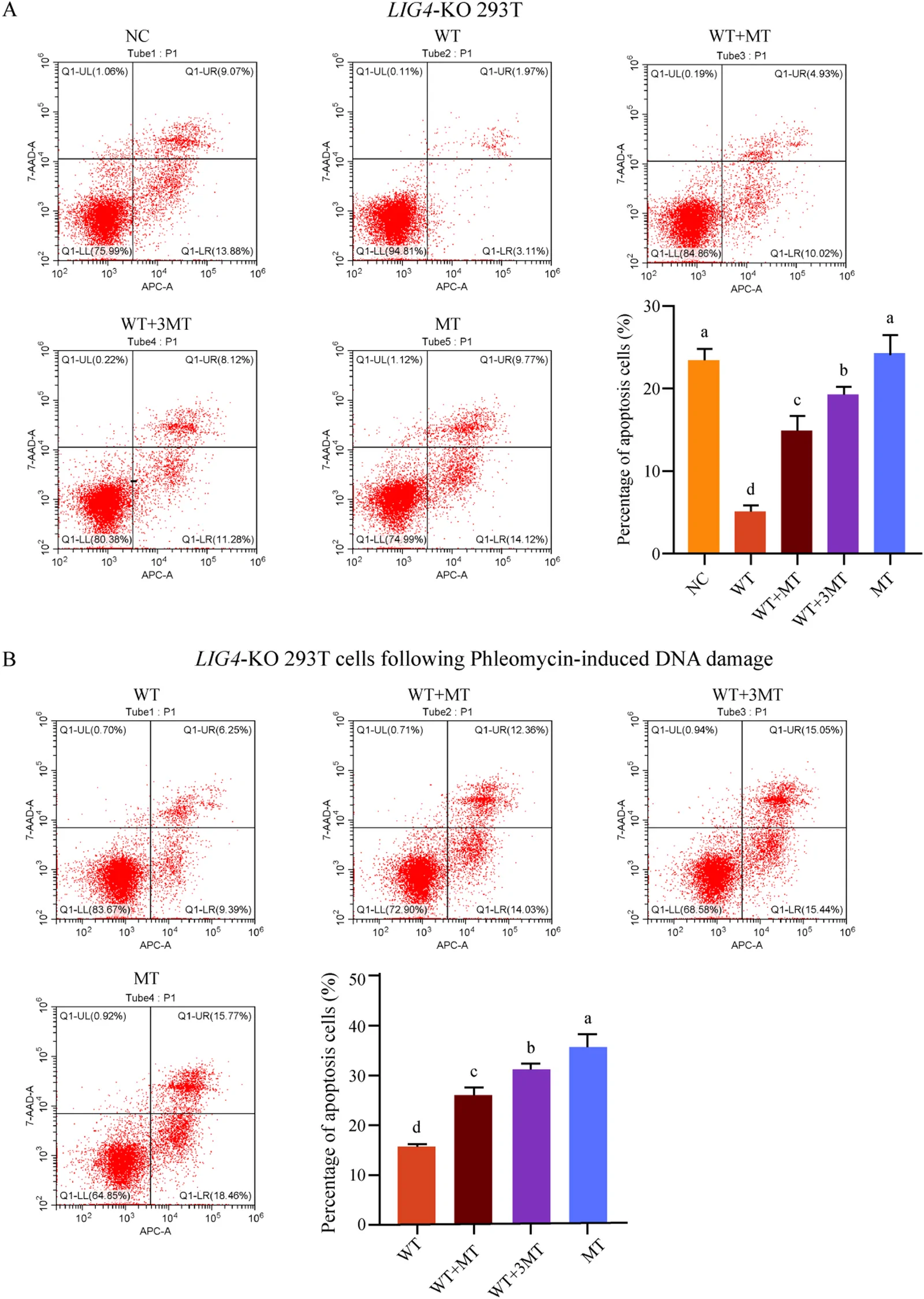
The *LIG4* p.Lys424ArgfsTer20 LoF variant manifests haploinsufficiency upon reconstitution in *LIG4*-KO 293T cells. **A** Basal apoptosis rates following transfection. *LIG4*-KO 293T cells were transfected with NC, WT, WT + MT (1:1), WT+3MT (1:3), or MT *LIG4* plasmids for 36 h, and the total apoptosis rates were assessed using flow cytometry. **B** Apoptosis rates following Phleomycin-induced DNA damage. Cells transfected with WT, WT + MT (1:1 and 1:3), or MT *LIG4* plasmids for 36 h were treated with phleomycin (50 µg/mL, 1 h) and allowed to recover in fresh media for 6 h prior to apoptosis assessment. Data are presented as mean ± SEM (*n* = 3 independent experiments). Statistical significance was determined using one-way ANOVA followed by Tukey’s post hoc test. Different letters indicate significant differences between groups (*P* < 0.05); groups sharing the same letter are not significantly different from each other

SiRNA knockdown reduces *LIG4* levels but cannot distinguish between haploinsufficiency and dominant-negative effects in heterozygous carriers. To differentiate between these mechanisms, we created a *LIG4*-null model (*LIG4*-KO 293T) and reconstituted cells with WT and MT *LIG4* plasmids at defined ratios, mimicking the heterozygous state while controlling the allele dosage. Apoptosis was quantified under basal conditions and following genotoxic challenges. Under basal conditions, five groups were assessed: NC (empty vector control), WT, WT + MT (1:1), WT+3MT (1:3), and MT *LIG4* plasmid only. Apoptosis was quantified using flow cytometry 36 h post-transfection. WT LIG4 significantly reduced apoptosis compared with that in the NC, whereas MT alone showed apoptosis rates comparable to that of the NC. These data support a loss-of-function effect of the mutant and do not support an intrinsic cytotoxic gain-of-function mechanism. In the co-expression groups, apoptosis increased in a mutant dose-dependent manner (WT < WT + MT < WT+3MT) but remained consistently lower than that in the MT-only group, indicating that WT LIG4 activity was preserved in the presence of the mutant protein (Fig. 4A).

To further test this under genotoxic stress, transfected cells were challenged with phleomycin (50 µg/mL, 1 h), allowed to recover for 6 h, and then assessed for their apoptotic status. Because basal-condition data showed that the NC and MT-only groups exhibited equivalent apoptosis rates, the MT-only group was used as the repair-deficient reference, representing the background level in the absence of effective *LIG4*-mediated repair. Following the phleomycin challenge, WT LIG4 conferred the strongest protection against apoptosis. Apoptosis rates increased progressively with the proportion of mutant plasmids (WT < WT + MT < WT+3MT < MT), and both co-expression groups displayed significantly lower apoptosis than the MT-only group (Fig. 4B). These results indicate that WT LIG4 retained its full protective capacity even when co-expressed with excess mutant LIG4 protein, consistent with a LoF mechanism in which the mutant allele neither interferes with nor augments the WT activity.

**Fig. 5:**
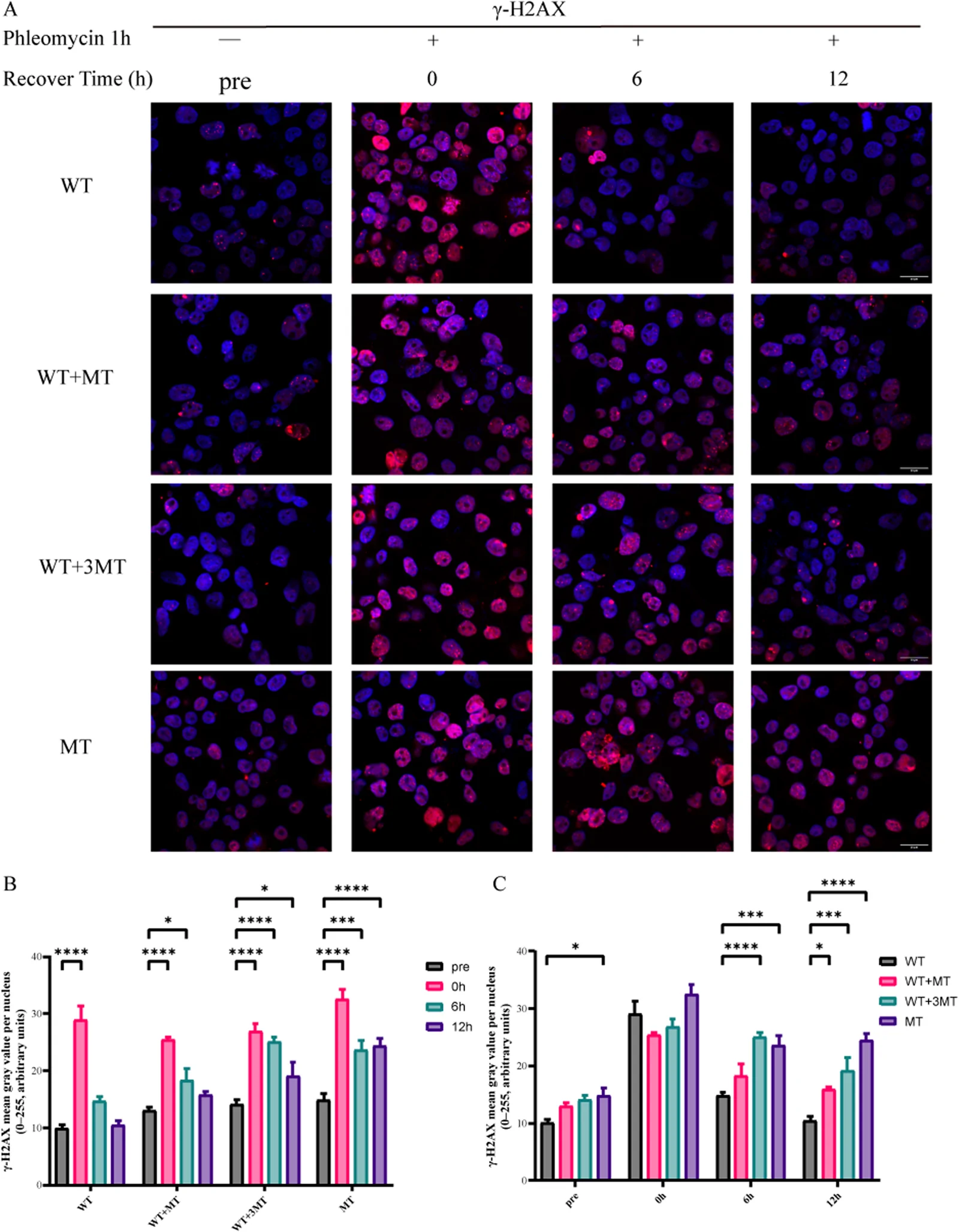
γH2AX clearance is progressively delayed with increasing mutant *LIG4* dosage in *LIG4*-KO 293T cells, consistent with haploinsufficiency. *LIG4*-KO 293T cells were transfected with WT, WT + MT (1:1), WT+3MT (1:3), or MT *LIG4* plasmids for 36 h, followed by phleomycin treatment (1 h). γH2AX levels were assessed before treatment (pre) and at 0, 6, and 12 h of recovery. **A** Representative immunofluorescence images of γH2AX. Red fluorescence indicates γH2AX foci; blue fluorescence (DAPI) indicates nuclei. Scale bar = 20 μm. **B** Quantification of the mean γH2AX fluorescence intensity (mean gray value) per nucleus at different recovery times. Statistical comparisons within each group were made against the pre-treatment time points. **C** Comparison of γH2AX fluorescence intensity per nucleus among groups at pre, 0, 6, and 12 h time points. Statistical comparisons were made with the WT group at each specific time point. Data are presented as mean ± SEM (*n* = 3 independent experiments). Statistical analysis was performed using two-way ANOVA, followed by Dunnett’s multiple comparisons test. *P < 0.05, ***P < 0.001, ****P < 0.0001

Next, we examined whether the dose-dependent pattern extended to DSB repair kinetics. *LIG4*-KO 293T cells were transfected with WT, WT + MT (1:1), WT+3MT (1:3), or MT *LIG4* plasmids. At 36 h post-transfection, DNA damage was induced with phleomycin (50 µg/mL, 1 h), and immunofluorescence was performed before treatment (pre) and at 0, 6, and 12 h after drug washout to quantify γH2AX (Fig. 5A) and 53BP1 levels (Figure S2). Two-way ANOVA revealed significant effects of time and treatment on γH2AX levels, indicating altered repair kinetics across *LIG4* configurations. At 0 h, γH2AX levels increased sharply and comparably across all groups relative to baseline (pre), indicating equivalent initial DNA damage loads. Thereafter, γH2AX levels rapidly declined in WT and WT + MT cells, returning to near baseline levels by 12 h. In contrast, the signals in WT+3MT cells remained elevated at 6 h and showed only a partial reduction at 12 h. In MT-only cells, γH2AX remained significantly above the baseline at both 6 and 12 h, with no appreciable clearance during this late phase (Fig. 5B). Between-group comparisons at each time point (Fig. 5C) showed that while MT cells had slightly higher basal damage at the pre-treatment stage, no differences were observed at 0 h. However, at 6 h, both the WT+3MT and MT groups exhibited significantly higher γH2AX levels than the WT group, and by 12 h, all three non-WT conditions retained significantly more γH2AX than the WT group. Overall, γH2AX clearance was progressively delayed in the following order: WT < WT + MT < WT+3MT < MT. Importantly, both co-expression groups consistently showed lower residual signals than the MT-only group. Together with the concordant 53BP1 dynamics (Figure S2), these data demonstrate that the variant causes a dose-dependent reduction in overall repair efficiency and delayed damage marker resolution, which is characteristic of a LoF allele. Furthermore, because co-expression with the mutant did not compromise the protective activity of the wild-type allele, these results effectively ruled out a dominant-negative effect.

### Spatiotemporal expression patterns and functional enrichment analysis of *LIG4* during human folliculogenesis

Having established that *LIG4* haploinsufficiency compromises DNA repair in granulosa cells, we next sought to delineate the physiological spatiotemporal expression landscape of *LIG4* within the human follicular niche using a single-cell RNA sequencing dataset (GSE107746).

**Fig. 6:**
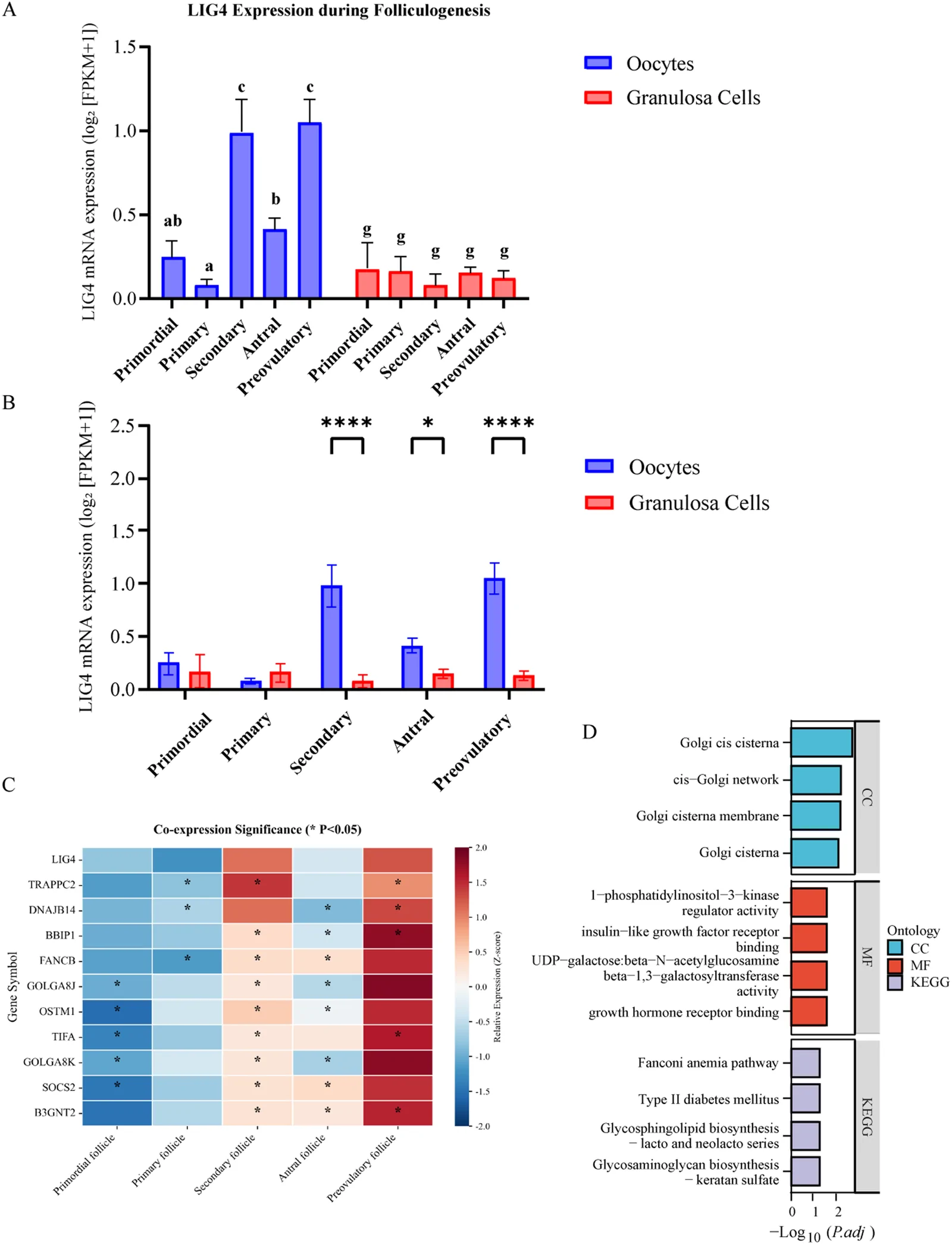
Spatiotemporal expression patterns and functional enrichment analysis of*LIG4*during human folliculogenesis. **A ***LIG4* mRNA expression (log₂[FPKM + 1]) in oocytes and granulosa cells across five developmental stages: primordial, primary, secondary, antral, and preovulatory follicles. Data are presented as mean ± SEM. Different lowercase letters above the bars indicate statistically significant differences between stages in oocytes (*P* < 0.05). The absence of different letters for granulosa cells indicates no significant difference between stages. **B** Comparative analysis of *LIG4* mRNA expression between oocytes (blue) and granulosa cells (red) at each developmental stage. Error bars represent the mean ± SEM. *P < 0.05, ***P < 0.0001. **C** Heatmap showing the expression profiles of the 10 core *LIG4* co-expressed genes across the five follicular stages. The expression levels were Z-score-normalized (blue: low expression; red: high expression). Asterisks indicate stages where the specific gene expression shows a significant positive correlation with *LIG4* (Pearson correlation, *P* < 0.05). **D** GO (CC and MF) and KEGG pathway enrichment analysis of the 10 identified co-expressed genes. The bar chart displays significantly enriched GO terms (CC: Cellular Component; MF: Molecular Function) and KEGG pathways (*P*_*adj*_*<0.05*)

Two-way ANOVA revealed that the developmental stage, cell type, and their interaction exerted highly significant effects on *LIG4* expression. Specifically, oocytes exhibited a “stepwise upregulation” pattern, with expression being the lowest in primary follicles, intermediate in primordial and antral follicles, and peaking at the secondary and preovulatory stages (with no significant difference between the latter two stages). In contrast, *LIG4* expression in granulosa cells remained consistently low and stable, with no significant variation across developmental stages (Fig. 6A). Comparative analysis further highlighted the stage-dependent divergence between these two cell types. While *LIG4* levels were comparable between oocytes and granulosa cells during the early stages (primordial and primary follicles), a significant divergence emerged in the later stages, where *LIG4* expression in oocytes significantly exceeded that in granulosa cells from the secondary to preovulatory stage (Fig. 6B). This pronounced expression disparity in the later developmental stages suggests a specialized and critical need for *LIG4* to maintain genomic integrity during oocyte maturation.

To delineate the potential regulatory network of *LIG4* in oocytes, we identified ten co-expressed genes (e.g., *FANCB*, *SOCS2*, and *TIFA*) that exhibited synchronous expression dynamics with *LIG4* across follicular development (Fig. 6C). GO and KEGG enrichment analyses of these co-expressed genes revealed distinct functional themes (Fig. 6D). In terms of Cellular Components (CC), the genes showed a distinct localization pattern associated with the Golgi apparatus, being significantly enriched in the Golgi cis cisterna, cis-Golgi network, and Golgi cisterna membrane. Regarding Molecular Functions (MF), enrichment was primarily observed in growth factor signaling and enzymatic regulation, specifically involving growth hormone receptor binding, insulin-like growth factor receptor binding, 1-phosphatidylinositol-3-kinase regulator activity, and UDP-galactose: beta-N-acetylglucosamine beta-1,3-galactosyltransferase activity. Furthermore, KEGG pathway analysis highlighted the functional networks associated with these genes. Notably, the Fanconi anemia pathway was significantly enriched, which is consistent with its role in DNA repair. The analysis also revealed enrichments in metabolic pathways, including Type II diabetes mellitus, as well as glycan biosynthesis pathways, such as glycosaminoglycan biosynthesis-keratan sulfate and glycosphingolipid biosynthesis-lacto and neolacto series.

## Discussion

In this Han Chinese family with non-syndromic POI, we identified a monoallelic *LIG4* frameshift truncation (c.1271_1275del; p. Lys424ArgfsTer20) that co-segregated with the phenotype. This variant is a canonical LoF allele that meets the ACMG PVS1 criteria. Although ClinVar predominantly associates *LIG4* variants with autosomal recessive LIG4 syndrome [16, 27, 28], our study suggests that *LIG4* haploinsufficiency may contribute to a pathogenic mechanism that extends beyond the established syndromic features to the field of reproductive biology.

We have summarized previously reported cases of *LIG4* deficiency associated POI in (Table 3). While hypergonadotropic hypogonadism/POI has been documented in specific biallelic cases (most compound heterozygous) [29–31], it is often overshadowed by severe multisystemic anomalies. In contrast, heterozygous carriers in our cohort displayed a much milder presentation. This stark contrast supports the “tissue-specific threshold model” for DNA repair. Our findings parallel recent reports that monoallelic *LIG4* variants drive isolated immune dysregulation [17]; together, these studies overturn the traditional view of *LIG4* deficiency as strictly recessive. Instead, they suggested that high-turnover tissues, specifically lymphoid and germline compartments, are uniquely vulnerable to haploinsufficiency. This biological pattern aligns with the phenotype of *NHEJ1* haploinsufficiency, which similarly restricts ovarian competence without causing systemic disease [18], reinforcing the concept that the entire NHEJ complex operates under strict dosage constraints in the human ovary.

**Table 3:** *LIG4* deficiency associated POI

Study Year	Age at examination	Allelic Status	Mutations	Gonadal Phenotype	Systemic Features
2014 [30]	17y6m	compound heterozygous	p. (Arg814^*^) + p. (Lys424Argfs^*^20)	POI	Small cerebral aneurysm, Mild developmental delay
2014 [30]	11y9m	compound heterozygous	p. (Arg814^*^) + p. (Lys424Argfs^*^20)	POI	Atrial ventricular septal defect, atrophic kidney, rib hypoplasia, fusion of carpal bones, copper beaten skull, platybasia, abnormal C1 vertebrae, mild moderate developmental delay
2019 [31]	18y	compound heterozygous	p.R814*+ p. S205Lfs*29	hypergonadotropic hypogonadism / POI	microcephaly, growth failure, mild pancytopenia, hepatocellular carcinoma
2025 [29]	15y	Biallelic (homozygous)	p. Arg814*	hypergonadotropic hypogonadism / POI	Seckel-like microcephaly, lissencephaly, intellectual disability, mild leukopenia

The pathogenicity of the specific *LIG4* variant identified (c.1271_1275del; p. Lys424Argfs*20) provides a molecular basis for this haploinsufficiency model. Although this well-established null allele is known to cause devastating systemic failure in biallelic carriers [32–37], our structural modeling and cellular assays revealed why a single hit is sufficient to compromise ovarian function. Structurally, the mutation truncates the C-terminal BRCT domain, which is essential for XRCC4 interaction. Because the LIG4-XRCC4 interaction is obligatory for ligase stability, disrupting this critical interface renders the truncated protein unstable and susceptible to degradation [38]. This creates a state of functional haploinsufficiency, which we confirmed in our cell model: partial *LIG4* depletion compromised the “repair threshold,” leading to delayed DNA repair kinetics and exacerbated stress-induced senescence. Our findings are further corroborated by the clinical phenotypes of LIG4 partners, such as *XRCC4*, where mutations are similarly associated with gonadal failure [39]. Collectively, these data suggest that the integrity of the entire LIG4-XRCC4-NHEJ1 ligation complex may be non-redundant and strictly dosage-dependent in maintaining human ovarian function.

While our cellular models confirmed the functional impact of *LIG4* haploinsufficiency in granulosa cells, understanding the physiological demand for *LIG4* within the follicular niche is necessary to explain the tissue-specific vulnerability of the ovary. Recent spatiotemporal transcriptomic analyses of human ovaries have identified the downregulation of DDR pathways as a key driver of oocyte aging [40]. To establish a healthy physiological baseline for *LIG4* expression during folliculogenesis, we reanalyzed the GSE107746 single-cell dataset, which provides an uninterrupted transcriptomic trajectory of wild-type human oocytes. Unlike granulosa cells, which maintain low basal *LIG4* expression, oocytes exhibit a pronounced bimodal trajectory, which surges specifically during the secondary and pre-ovulatory stages. This dynamic aligns with the global transcriptional silencing that occurs during oocyte meiotic maturation (from the germinal vesicle to the metaphase II stage) [41, 42]. The pre-ovulatory peak likely reflects a maternal storage strategy to accumulate *LIG4* transcripts, which are necessary to manage the intense DSB pressure accompanying meiotic resumption in oocytes. Crucially, this peak demand exposes developmental bottlenecks. While somatic cells can often compensate for defective NHEJ via homologous recombination (HR) [43], HR is physiologically suppressed during oocyte maturation to prevent aberrant crossovers and ensure precise chromosome segregation [44]. This suppression leaves pre-ovulatory oocytes exclusively reliant on the NHEJ pathway for DNA repair. In heterozygous carriers, *LIG4* haploinsufficiency creates a severe dosage deficit when demand peaks. Without alternative repair networks, unresolved DSBs trigger checkpoint-mediated apoptosis or developmental arrest, providing a clear physiological basis for the accelerated follicular depletion and secondary amenorrhea observed in these patients.

Beyond mere abundance, our co-expression analysis positioned *LIG4* as a central node in a high-fidelity surveillance network. First, the synchronous upregulation of *LIG4* with *FANCB* (Fanconi Anemia Complementation Group B), a key component of the FA core complex, aligns with recent findings that variants in the related gene *FANCI* cause preovulatory failure, suggesting a cooperative alliance between the NHEJ and FA pathways during meiotic maturation [11]. Second, the enrichment of insulin-like growth factor (IGF) binding and co-expression with SOCS2, a critical negative regulator of GH/IGF-1 signaling, links DNA repair to metabolic regulation. This molecular signature strikingly mirrors the phenotype of *XRCC4* deficiency, where patients present with gonadal failure, hypothyroidism, diabetes, dyslipidemia, and severe growth defects [39], reinforcing the concept that the LIG4-XRCC4 complex serves as a critical molecular hub coupling genomic surveillance with metabolic growth checkpoints in the oocyte. Finally, co-expression with *TIFA* (*TRAF*-interacting protein with a forkhead-associated domain), a DNA damage-sensing adaptor involved in innate immunity [45], suggests that unresolved DNA lesions may trigger immune-mediated follicular atresia. Thus, this suggests that *LIG4* haploinsufficiency may compromise not only its direct enzymatic activity but also implicates it in a coordinated network crucial for oocyte competence.

### Limitations

This study has several limitations. Although our genetic and functional data are robust, they were derived from a single pedigree. Given the extreme genetic heterogeneity of non-syndromic POI, future multicenter screenings using larger cohorts are necessary to determine the broader epidemiological prevalence of *LIG4* variants. Furthermore, while our in vitro models clarified the cellular consequences of haploinsufficiency, they could not fully replicate the ovarian microenvironment. Consequently, the ovarian-specific conclusions proposed in this study have not been directly tested in vivo and remain to be validated. Since global *Lig4* knockout in mice is embryonic lethal [46], its in vivo impact on reproductive aging remains unknown [47, 48]. Future studies employing oocyte-specific conditional knockouts and integrated reporter systems (e.g., EJ5-GFP) are essential for directly quantifying NHEJ efficiency in vivo. Finally, our single-cell transcriptomic reanalysis provides a valuable temporal context but remains inherently correlative.

### Potential translational perspectives

Uncovering the precise genetic etiology of POI may eventually inform clinical perspectives that extend beyond basic diagnostics, potentially facilitating a shift from reactive hormone replacement to proactive risk management [49]. Because *LIG4* deficiency inherently heightens broader genomic instability and malignancy risks [50], an important question for future research is whether premature ovarian aging in these patients could serve as an early clinical sentinel. This highlights the conceptual importance of investigating whether tailored oncological surveillance and minimizing exposure to ionizing radiation are warranted for such carriers.

Beyond risk surveillance, pinpointing the specific genetic cause may eventually guide strategies for reproductive interventions. While studies show that a confirmed genetic diagnosis can help predict the remaining ovarian reserve in approximately 60.5% of POI patients [51], whether this applies to *LIG4* carriers to identify individuals who could benefit from early fertility preservation before follicular exhaustion remains unclear. From a pharmacological perspective, as the field explores gerotherapeutics for reproductive aging [52], this haploinsufficient *LIG4* deficiency could be theoretically mitigated through dosage-restoration strategies. As reviewed by Evans et al. [53], tools such as antisense oligonucleotides or small-molecule potentiators could be explored to restore LIG4 protein levels and maintain the ovarian function. Additionally, targeted antioxidant therapies may alleviate baseline genotoxic stress and prevent oocyte apoptosis [54]. Notably, our single-cell data revealed a stage-specific peak in *LIG4* expression during meiotic resumption, a period associated with surging endogenous double-strand breaks. This dynamic suggests that targeted prophylactic treatments during specific windows, such as preceding ovulation or during IVF cycles, represent an intriguing avenue for future studies to help vulnerable oocytes cross the damage threshold. However, considering that our findings are based on a single-family observation and in vitro models, direct clinical translation remains premature. Therefore, the proposed dosage-restoration therapies, malignancy surveillance protocols, and lifestyle recommendations to minimize radiation exposure should be strictly viewed as hypothesis-generating future directions that require rigorous clinical and functional validation before being considered as actionable interventions.

## Conclusion

In conclusion, we identified a monoallelic loss-of-function variant of *LIG4* in a Han Chinese family with non-syndromic POI. Functional assays demonstrated that *LIG4* haploinsufficiency markedly impairs DNA repair capacity, predisposing granulosa cells to stress-induced apoptosis and senescence. Human single-cell transcriptomic profiling further highlights the indispensable role of *LIG4* in maintaining genomic integrity during pre-ovulatory oocyte maturation. These clinicogenomic and experimental findings indicate that monoallelic *LIG4* mutations represent a potential ultra-rare genetic etiology of non-syndromic POI with sex-limited penetrance.

## Supplementary information

Supplementary Material 1.

## Data Availability

The single-cell RNA sequencing dataset analyzed in this study is publicly available in the Gene Expression Omnibus (GEO) database under the accession number GSE107746. WES in this study was performed in a very small family cohort (two individuals). Owing to ethical and privacy considerations related to human genetic data, the raw sequencing data are not publicly available but can be provided by the corresponding author upon reasonable request. Other data generated or analyzed during this study are included in this article and its supplementary information files.

## Abbreviations

POI: Primary ovarian insufficiency
FSH: Follicle-stimulating hormone
WES: Whole exome sequencing
LoF: Loss-of-function
DDR: DNA damage response
HR: Homologous recombination
DSBs: Double-strand breaks
GWAS: Genome-wide association studies
GCs: Granulosa cells
PGCs: Primordial germ cells
NHEJ: Non-Homologous End Joining
LIG4: DNA Ligase IV
CDS: Coding sequence
ANOVA: One-way analysis of variance
DBD: DNA-binding domain
OBD: Oligonucleotide-binding domain
AdD: Adenylation/activation domain
BRCT: BRCA1 C-terminal repeat
FANCB: Fanconi Anemia Complementation Group B
IGF: Insulin-like growth factor
SOCS2: Suppressor of Cytokine Signaling 2
TIFA: TRAF interacting protein with forkhead associated domain
MF: Molecular Function
CC: Cellular Component
GO: Gene Ontology
KEGG: Kyoto Encyclopedia of Genes and Genomes
