# Supplementary material for: Non-syndromic premature ovarian insufficiency associated with monoallelic LIG4 mutation via haploinsufficiency
Source: J Ovarian Res. 2026 Jun 24;19:281. doi: 10.1186/s13048-026-02163-1 (PMC13556010; doi:10.1186/s13048-026-02163-1)
Supplement: Supplementary file 1 [file 13048_2026_2163_MOESM1_ESM.docx]

**Figure S1 Predicted secondary structure of wild-type and mutant LIG4 protein.** (A-B) SOPMA analysis of wild-type (A) and p. Lys424ArgfsTer20 mutant (B) LIG4, showing an increase in α-helix content (blue) and a decrease in β-sheet content (red) in the mutant. (C-D) PSIPRED analysis of wild-type (C) and mutant (D) LIG4 (α-helix s are shown in pink, and β-sheets are in yellow). The concordant results highlight the loss of C-terminal structural modules and the remodeling of secondary elements near the truncation site.

**Figure S2 Figure 5 Assessment of DNA damage repair kinetics in *LIG4*-KO 293T cells with different WT/MT LIG4 plasmid ratios.** *LIG4*-KO 293T cells were transfected with WT, WT+MT (1:1), WT+3MT (1:3), or MT LIG4 plasmids for 36 h, followed by Phleomycin treatment (1 h). 53BP1 levels were assessed before treatment (pre) and at 0, 6, and 12 h of recovery. (A) Representative immunofluorescence images of 53BP1. Red fluorescence indicates 53BP1 foci; blue fluorescence (DAPI) indicates nuclei. Scale bar = 20 μm. (B) Quantification of the mean 53BP1 fluorescence intensity (mean gray value) per nucleus at different recovery time points. Statistical comparisons within each group were made against the pre-treatment time point. (C) Comparison of 53BP1 fluorescence intensity per nucleus among groups at pre, 0, 6, and 12 h time points. Statistical comparisons were made against the WT group at each specific time point. Data are presented as mean ± SEM (n = 3 independent experiments). Statistical analysis was performed using two-way ANOVA followed by Dunnett’s multiple comparisons test. **P* < 0.05, ****P* < 0.001, *****P* < 0.0001.
